# Respiratory syncytial virus as a cause of pulmonary hemorrhage in a low birth weight infant - strategies for protection and prevention: a case report

**DOI:** 10.4076/1757-1626-2-7455

**Published:** 2009-06-09

**Authors:** Shetal Shah, Martha Caprio

**Affiliations:** 1 Division of Neonatal Medicine, Department of Pediatrics, Stony Brook University School of MedicineHealth Science Tower 11-060, Stony Brook, NY 11794USA; 2 Division of Neonatal Medicine, Department of Pediatrics, New York University Medical Center, Tisch Hospital/Bellevue Medical Center560 First Avenue, New York, NY 10010USA

## Abstract

**Introduction:**

Respiratory Syncytial Virus is a common neonatal pathogen. Here we present a case of a premature, low birth weight infant who contracted respiratory syncytial virus and developed a severe pulmonary hemorrhage.

**Case presentation:**

A 12-day-old Asian male, former 30-week premature infant with a birth weight of 1025 grams presented with nasal secretions, episodes of desaturations and increased work of breathing. The infant developed a pulmonary hemorrhage. Secretions during tracheal lavage were positive for respiratory syncytial virus on rapid fluorescence assay. After supportive care, the patient improved. Isolation, cohorting techniques and reinforcement of strict hand-washing guidelines prevented and outbreak to other infants.

**Conclusion:**

This original case report presents an uncommon presentation of respiratory syncytial virus infection, a common pediatric pathogen. Neonatologists should consider evaluating patients with pulmonary hemorrhage for respiratory syncytial virus if preceding symptoms are consistent with that infectious illness.

## Introduction

Respiratory Syncytial Virus (RSV) is a potentially lethal pathogen in the pediatric population [1]. Particularly affected are premature infants less than six months old. RSV is a major cause of apnea, respiratory distress, respiratory failure and obstructive lung disease [2,3]. We are the first to describe RSV and an association for acute pulmonary hemorrhage in a 1000 gram premature infant. We further review mechanisms and evidence for containing the spread of this pathogen upon detection in the neonatal intensive care unit (NICU).

## Case presentation

Our patient, a 12-day-old Asian male infant, weighed 1025 grams and was the second twin born of in-vitro fertilized donor eggs to a 47-year-old nulliparous woman at 30^1^/_7_ weeks gestation. The pregnancy was complicated by hypertension requiring 10 days of hospitalization prior to delivery for administration of labetolol. Her condition was associated with normal liver function tests and a platelet count of 150,000/ mcl. Prior to delivery, the mother received dexamethasone and ampicillin therapy because of the unknown status of her group *B streptococcus* cervical cultures. Premature spontaneous vaginal delivery (ascribed to hypertension and mild toxemia) was complicated with breech presentation and poor respiratory effort requiring 30 seconds of positive-pressure ventilation. Apgar scores were 5 at one minute and 7 at five minutes. The infant was intubated for worsening respiratory distress at 20 minutes of life. Initial arterial blood gas demonstrated a pH of 7.35, carbon dioxide of 44 mmHg, PaO2 of 66 mmHg with a bicarbonate level of 23 mg/dl and a base deficit of -1.6. Beractant was delivered via endotracheal tube at 1.5 hours of life. Echocardiography revealed a 2 mm patent ductus arteriosis on the first day of life treated with 72 hours of indomethicin. Repeat echocardiography demonstrated closure of the ductus. Feeds were begun, via naso-gastric tube, on the third day of life. Antibiotic therapy with ampicillin and gentamicin were continued for a total of seven days for presumed bacteremia. Complete blood count at birth was not significantly abnormal revealing a WBC count of 7,500 /mcl, a hematocrit of 53%, however platelets were low at 132,000 and were attributed to the low maternal platelet count. Initial immature to total neutrophil ratio on admission was 0.2. Initial cranial ultrasound was normal for gestational age.

**Figure 1.**
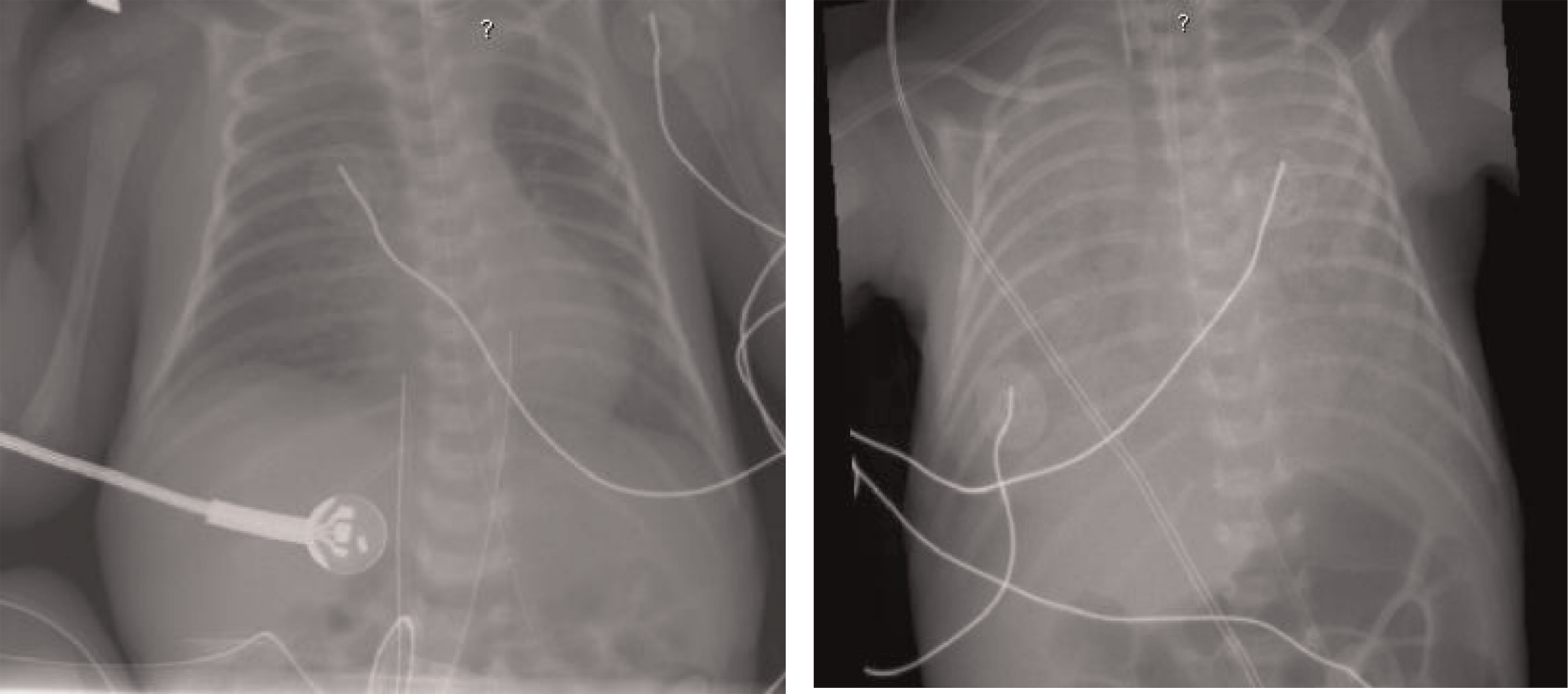
Chest X-ray: Left panel: The patient's chest X-ray the evening before demonstrating a coarse lung fields without diffuse or focal infiltrates consistent with respiratory distress syndrome. Right panel: Immediately after acute respiratory compensation, diffuse areas of infiltrates encompassing the entire lung fields consistent with diffuse pulmonary hemorrhage.

The patient weaned from conventional mechanical ventilation and was extubated to nasal continuous positive airway pressure of 5 cm H_2_O on the second day of life. He required only nasal cannula supplementation by his seventh day of life. On the twelfth day of life, at a weight of 1045 grams, the infant presented with increasing nasal secretions and oxygen desaturation episodes. Within 45 minutes the patient's work of breathing increased and his respiratory rate rose to 100 breaths/minute requiring intubation. Large amounts of fresh blood were noted upon laryngoscopy prior to intubation. Extensive bleeding was noted after endotracheal tube placement with worsening respiratory function requiring increased ventilatory settings in 100% Fio2 to maintain pulse oximetry saturations of 87%. Arterial blood gas and complete blood count sampling at this time were significant for a pH of 6.99, carbon dioxide of 74 mmHg, paO2 of 48 mmHg with a base deficit of -15. The infant's hematocrit decreased from 30.1% the previous day to 19% with a platelet count of 252,000. Mean arterial pressure decreased from a baseline of 33 mmHg to 27 mmHg with associated tachycardia to 220 beats per minute. Partial thromboplastin time and prothrombin time at this time were normal for gestational age. Chest X-Ray (Figure 1) was consistent with acute pulmonary hemorrhage.

After placement on high frequency oscillatory ventilation (HFOV) as well as three rapid infusions of packed red blood cells, the infant stabilized. Treatment with vancomycin, cefotaxime and acyclovir were initiated. Blood culture and surface cultures for herpes simplex virus were negative. The possibility of RSV was entertained upon discussion with the nurse caring for the infant. She noted the patient's father engaged in kangaroo care with the infant and his twin while exhibiting clear rhinorrhea and sneezing. Rapid RSV and influenza studies were sent from both nasal secretions and tracheal aspirates. Rapid RSV was positive in both specimens and RSV was also demonstrated from the father's secretions via rapid assay. Antibiotics were discontinued after 48-hours of blood cultures demonstrating no growth. Infectious disease consultation did not recommend use of ribavirin [4,5]. Nasal viral cultures and viral electron microscopy were positive for RSV but negative for the presence of other viruses.

The patient remained on HFOV for three additional days prior to weaning to conventional ventilation for 15 additional days. Head ultrasonography on day of life 19 demonstrated increased echogenicity in the left periventricular white matter consistent with cerebral ischemia as compared to a normal initial study obtained at seven days of age. The patient was discharged on hospital day 147.

## Discussion

RSV can be a major contributor to morbidity and mortality once in the NICU [6]. Once infection of host cells occurs, epithelial cell necrosis leads to the production of mucus plugs combined with cellular debris and subsequent inflammation, creating a one-way valve mechanism for air entry and localized atelectasis, leading to hyperinflation [3]. With decreased immune function, narrow alveolar diameters, decreased lung volume and underdeveloped lung anatomy; premature infants are uniquely susceptible hosts to this pathogen [6].

Though the major clinical manifestations of RSV include increased secretions associated with obstructive-type lung disease, apnea, wheezing and respiratory distress, we report the first case of pulmonary hemorrhage associated with RSV infection in an otherwise stable low birth weight infant. Risk factors for pulmonary hemorrhage in neonates include prematurity, mechanical ventilation, bleeding diathesis, patent ductus arteriosis, exogenous surfactant administration, and barotrauma. Given the multi-factorial nature of pulmonary hemorrhage and the presence of risk factors in this infant, direct causality of RSV cannot be determined.

However, the patient's negative blood cultures, failure to improve on antibiotics, negative viral cultures for other viruses such as adenovirus, normal coagulation profile and positive RSV assay argue for this agent as a probable factor for pulmonary hemorrhage, especially in the context of a known sick contact. Further studies are required to further clarify the relationship between these two conditions.

Numerous outbreaks of RSV in the NICU have been described [7-9]. Given its high degree of infectivity and ability to survive on inanimate surfaces, the NICU allows for efficient spread of the virus to other neonates, especially if strict hand washing policies are not enforced. Thus once an index case is discovered, it is critical to prevent spread of the pathogen and secondary cases. Currently, American Academy of Pediatric (AAP) guidelines for control of nosocomial RSV infection is strict adherence to infection control practices, including:

1. Strict handwashing policies before and after each patient contact for 15 seconds.
2. Laboratory screening of patients for RSV infection.
3. Cohorting infected patients and staff.
4. Exclusion of visitors and staff with respiratory tract infections.
5. Use of gowns, gloves goggles and masks.

These guidelines were directed at the older age high-risk pediatric hospital population, not the closed environment of the NICU. Kilani described a stricter infection control policy during eight cases of RSV in the neonatal unit. Affected patients were cohorted and examined only with gowns, gloves and masks in accordance with the guidelines established by the AAP for older children [7]. While anecdotal, such policies should also be considered in the NICU population.

Once detection of RSV had occurred in our unit, all infants cared for by the patient's nurse for the past 10 days were tested for RSV infection. RSV infection was detected in two other infants, including the patient's brother, who was symptomatic with rhinorrhea and coughing. The patients were cohorted and assigned one nurse with strict contact precautions observed. No specific medical team was assigned to these three patients and the NICU was not fumigated. Palivizumab, was administered to all infants meeting the AAP pre-discharge and outpatient criteria for preventive administration after surveillance testing documented the patients as RSV negative. Additional discussion with families and staff was enforced regarding the importance to restrict visitation by individuals with respiratory infections. No further cases were identified.

## Conclusions

While there is no controlled study on the use of palivizumab for the prophylaxis of NICU patients during an outbreak of RSV in the NICU, this case adds to anecdotal evidence such measures should be considered upon detection of secondary cases of RSV [10]. As infection control measures are now standard of care for the prevention of nosocomial RSV and outbreaks in the NICU tend to consist of a small number of patients, it is doubtful a controlled study with significant statistical power will be undertaken soon, making the exact benefit of palivizumab administration in this setting difficult to define.

## List of abbreviations

AAP: American Academy of Pediatrics
HFOV: High frequency oscillatory ventilation
NICU: Neonatal Intensive Care Unit
RSV: Respiratory Syncytial Virus
WBC: White blood cells
